# Impact of pre-imputation SNP-filtering on genotype imputation results

**DOI:** 10.1186/s12863-014-0088-5

**Published:** 2014-08-12

**Authors:** Nab Raj Roshyara, Holger Kirsten, Katrin Horn, Peter Ahnert, Markus Scholz

**Affiliations:** 1 Institute for Medical Informatics, Statistics and Epidemiology, University of Leipzig, Haertelstrasse 16-18, Leipzig, 04107, Germany; 2 LIFE Center (Leipzig Interdisciplinary Research Cluster of Genetic Factors, Phenotypes and Environment), University of Leipzig, Philipp-Rosenthal Strasse 27, Leipzig, 04103, Germany; 3 Department for Cell Therapy, Fraunhofer Institute for Cell Therapy and Immunology, Perlickstrasse 1, Leipzig, 04103, Germany; 4 Translational Centre for Regenerative Medicine, Universität Leipzig, Philipp-Rosenthal-Strasse 55, Leipzig, 04103, Germany

**Keywords:** Genotype imputation, Pre-imputation filtering, SNP quality control, Genome-wide association analysis, SNP data

## Abstract

**Background:**

Imputation of partially missing or unobserved genotypes is an indispensable tool for SNP data analyses. However, research and understanding of the impact of initial SNP-data quality control on imputation results is still limited. In this paper, we aim to evaluate the effect of different strategies of pre-imputation quality filtering on the performance of the widely used imputation algorithms MaCH and IMPUTE.

**Results:**

We considered three scenarios: imputation of partially missing genotypes with usage of an external reference panel, without usage of an external reference panel, as well as imputation of completely un-typed SNPs using an external reference panel. We first created various datasets applying different SNP quality filters and masking certain percentages of randomly selected high-quality SNPs. We imputed these SNPs and compared the results between the different filtering scenarios by using established and newly proposed measures of imputation quality. While the established measures assess certainty of imputation results, our newly proposed measures focus on the agreement with true genotypes. These measures showed that pre-imputation SNP-filtering might be detrimental regarding imputation quality. Moreover, the strongest drivers of imputation quality were in general the burden of missingness and the number of SNPs used for imputation. We also found that using a reference panel always improves imputation quality of partially missing genotypes. MaCH performed slightly better than IMPUTE2 in most of our scenarios. Again, these results were more pronounced when using our newly defined measures of imputation quality.

**Conclusion:**

Even a moderate filtering has a detrimental effect on the imputation quality. Therefore little or no SNP filtering prior to imputation appears to be the best strategy for imputing small to moderately sized datasets. Our results also showed that for these datasets, MaCH performs slightly better than IMPUTE2 in most scenarios at the cost of increased computing time.

## Background

Imputation of missing genotype data is routinely used in current genetic data analyses. Here, we focus on the pre-imputation filtering process of SNPs which can be measured conveniently by many micro-array products or by sequencing techniques. There are three major scenarios in which imputation is usually applied: First, imputation can be used to fill the gaps of missing genotypes or to correct for genotyping errors in a self-content SNP dataset without an external reference panel (“hole filling without an external reference panel”). The second scenario is similar to the first, but a reference panel is used during the imputation process (“hole filling with an external reference panel”). The third scenario concerns with imputation of SNPs un-typed in all individuals (“entire SNP imputation”). Here, an external reference panel is mandatory. The latter scenario is typically relevant in genome-wide meta-analysis in order to combine datasets of different genotyping platforms comprising different subsets of SNPs. Another popular application for this scenario is to impute additional markers not available at any genotyping platforms, e.g. those retrieved from sequencing data. It has been shown that this approach might increase the power of genome-wide association studies [1],[2]. Prominent reference panels such as HapMap [3] and 1000 Genomes [4] are available for imputation purposes in different ethnicities.

A number of tools for genotype imputation were developed in the past such as MaCH [5], IMPUTE1 and IMPUTE2 [6],[7], BIMBAM [8], BEAGLE [9]–[11], and PLINK [12]. Implemented algorithms are based on modelling the haplotype structure of the population in different ways. A review of imputation software, implemented methods and comparisons of performance can be found in [13],[14]. While performing genotype imputation with any of these programs, an unavoidable biometrical question is how to deal with markers of low genotyping quality. Before analyzing any association with genetic markers, genotype data are usually filtered by a sequence of quality control (QC) steps. Typical criteria for filtering at SNP level are *low call rate (CR)*, concordance with *Hardy-Weinberg equilibrium (HWE)* and *low minor allele frequency (MAF)*. Current practice suggests that reliable statistical inference of SNPs can be achieved through imputation after removing bad quality SNPs (and individuals) from a given dataset [15]–[19]. Commonly used cut-offs for the SNP filtering criteria are [18],[20],[21]: MAF > 1–5%, HWE p-value > 10^−6^ - 10^−4^, and SNP call rate >90–99%. However, there is no common agreements regarding the cut-offs; and those recommendations mainly result from the standards used in genetic association analysis, but not from a dedicated analysis related to the impact of SNP-quality filtering on the imputation result. Indeed, filtering of genotypes may reduce accuracy of imputation results. Imputation algorithms typically exploit the linkage disequilibrium (LD) structure between markers, and consequently, imputation accuracy depends on the strength of LD between missing and available genotypes [10]. When filtering SNPs before imputation, LD structure between markers is thinned down. Hence, the relative merit of pre-imputation filtering of low quality SNPs is still debatable.

Southam et al. [22] recently suggested that imputation of common variants is rather robust to genotype quality. However, this conclusion was drawn on the basis of analyses performed only for a single imputation software and limited to the scenario “entire SNP imputation”.

In the present paper, we aim to fill this gap assessing the impact of SNP quality control on imputation accuracy for IMPUTE2 and the software MaCH. Both programs are among the most frequently applied imputation software. Additionally, we introduce and apply two new scores to assess imputation quality with improved characteristics. Most currently available imputation quality scores are defined only at SNP-wise level, therefore, they are of limited use for comparisons at genotype level. An existing measure at genotype level is to compare concordance of the best-guess imputed genotype with the known genotype. However, this does not take into account the posterior probabilities of imputed genotypes. As another disadvantage, most currently available measures are specific for the imputation software used. To overcome all these limitations, we defined new scores applicable at genotype level, which are platform independent and which can take posterior probabilities into account. Finally, we consider imputation scenarios not addressed before [22], including hole filling with and without an external reference panel.

## Methods

### Data sets

We studied 100 German individuals collected in a close area in Saxony and Thuringia. Individuals are a subset of a cohort of an ongoing study regarding genetics of dyslexia [23],[24]. 65 individuals were males. Ethical approval was obtained from the Ethics Committee of the University of Leipzig. The regional school council Leipzig approved access to study participants in schools. Informed and written consent was obtained from each parent. Individuals were genotyped using Genome-Wide Human SNP Array 6.0 (Affymetrix, Inc., Santa Clara, California, USA). Genomic DNA from these individuals was extracted from blood and saliva using standard silica-based methods and extraction as described by the manufacturer (DNA Genotek, Ottawa, Ontario, Canada and Qiagen, Hilden, Germany), respectively. Integrity of genomic DNA was verified applying agarose gel electrophoresis. Array processing was carried out as a service by the genome analysis centre (Helmholtz-Zentrum München, Munich, Germany). Genotypes were called using the birdseed version 1 algorithm [24] implemented in the Affymetrix Genotyping Console software version 4.0, with standard settings. Genotype calling was improved by including additional reference individuals. Overall call rate was between 94.6% and 99.3% with a mean and median call rate of 98.3% and 98.45%, respectively. Included samples passed all technical array-wide quality control criteria as implemented in Genotyping Console (Bounds, Contrast QC, Contrast QC (Random), Contrast QC (Nsp), Contrast QC (Nsp/Sty Overlap), and Contrast QC (Sty) had to be larger than 0.4).

Only unrelated individuals were studied, i.e. it holds that p-Hat < 0.05 for all pairs of individuals as calculated by PLINK [12] on the basis of our genome-wide data. Analysis of population stratification was based on 30,501 independent SNPs. Applying the EIGENSTRAT method [25] revealed no evidence for population stratification. Clustering of first principal components of our samples resulted in a homogenous distribution which partly overlaps with those of HapMap individuals of Caucasian descendant (HapMap CEU, Additional file 1: Figure S1). Fst indicated close relation between HapMap CEU and our sample (Fst = 0.00062, calculated with software Arlequin 3.5.1.3 [26]).

### SNP data subsets used for analysis

**Figure 1:**
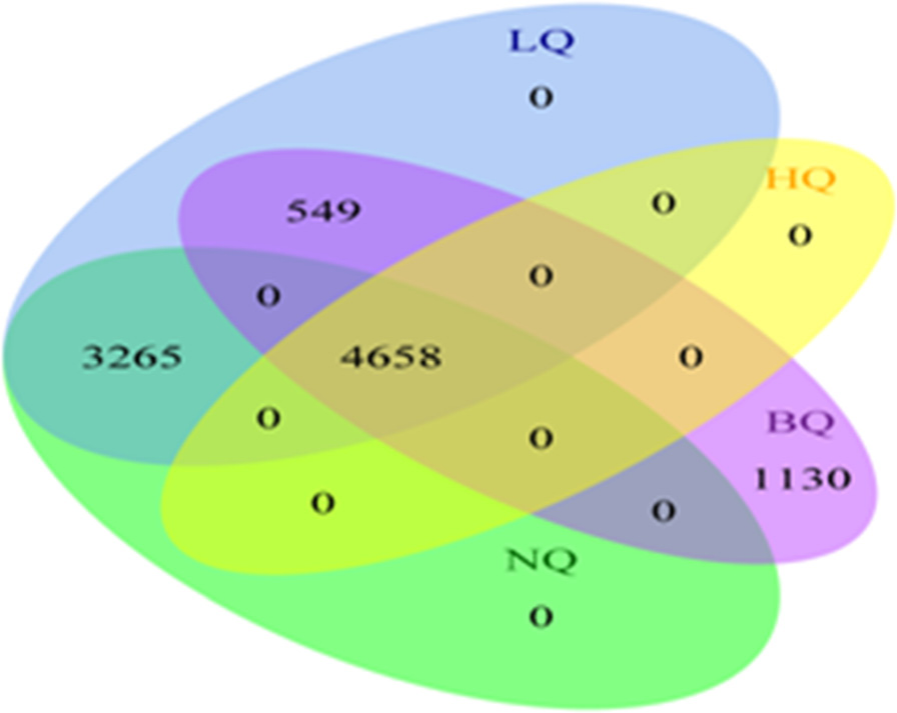
**Venn-Diagram describing the intersection of SNP datasets filtered by different quality criteria.** Note that by definition, HQ is contained in every subset.

All SNPs (in total 9,602) genotyped on chromosome 22 were studied. SNP data subsets with variable SNP quality were defined in order to test their performance in the imputation process. Based on various levels of MAF, CR and p-values of HWE test (as calculated by PLINK), we defined three basic subsets based on quality-filtering criteria namely: *high quality (HQ), normal quality (NQ), low quality (LQ)*: Subset ‘HQ’ was generated using highly stringent HQ-criteria: MAF ≥ 0.1, CR = 1, and p(HWE) ≥ 0.01. For these SNPs, we assume a high confidence of genotype calling. This judgment is based on published data [27] and on our own investigations (unpublished data) showing that the probability of genotyping error is less than 1% for these SNPs. The main approach to assess imputation quality in our study is first to mask a certain percentage of HQ genotypes, and then to compare the masked genotypes with corresponding results of the imputation process. Data subset NQ was created according to the recommendation of MaCH developers [16]. Here, SNPs were filtered by applying the criteria: MAF ≥ 0.01, CR ≥ 0.95, and p(HWE) ≥ 1×10^−6^. These criteria are also often applied in various GWA studies [15],[20],[21] for pre-imputation filtering process. The criteria used to create LQ-subset was defined by further relaxing the NQ-criteria: MAF > 0.005, CR ≥ 0.5, and p(HWE) ≥ 1×10^−12^. Data subset BQ was constructed by enriching SNPs of particularly low genotyping quality. It consists of all SNPs disqualified by the NQ criteria and all HQ SNPs. Finally, we called the scenario without any type of filtering as “ALL”. It is worthy to mention here that HQ SNPs were included in all of our data subsets which allows us comparing imputation results on the basis of this overlapping set of highly confident genotypes. Intersections of defined SNP datasets are illustrated as Venn-Diagram in Figure 1.

In order to investigate the impact of filtering with single quality criteria, we also considered scenarios based on the application of just one or two of the above mentioned criteria of MAF, CR and p(HWE). The results of these scenarios are presented as supplement material. In summary, a total of 16 scenarios were considered (Table 1).

**Table 1:** Description of scenarios of pre-imputation SNP filtering: Note that datasets contain different numbers of SNPs

**Data subset**	**Number of SNPs**	**Quality criteria for SNPs contained in the data subsets**
**HQ**	4658	high quality : criteria MAF ≥ 0.1, *CR* = 1 and p(HWE) ≥ 10^− 2^
**NQ**	7923	Normal quality : MAF ≥ 0.01, *CR* ≥ 0.95 and p(HWE) ≥ 10^− 6^
**LQ**	8472	low quality: MAF ≥ 0.005, *CR* ≥ 0.5, p(HWE) ≥ 10^− 2^
**NQ**.MAF	8310	MAF ≥ 0.01
**NQ**.HWE	9547	p(HWE) ≥ 10^− 6^
**NQ**.CAR	9194	*CR* ≥ 0.95
**HQ**.MAF	6344	MAF ≥ 0.1
**HQ**.HWE	9450	p(HWE) ≥ 10^− 2^
**HQ**.CAR	7148	*CR* = 1
**NQ**.MAF.HWE	8255	MAF ≥ 0.01_,_ p(HWE) ≥ 10^− 6^
**HQ**.MAF.HWE	6261	MAF ≥ 0.1_,_ p(HWE) ≥ 10^− 2^
**LQ**.MAF	8520	MAF ≥ 0.005
**LQ**.HWE	9574	p(HWE) ≥ 10^− 12^
**LQ**.MAF.HWE	8492	MAF ≥ 0.005_,_ p(HWE) ≥ 10^− 12^
**BQ**	6337	This data subset contains SNPs which fail NQ criterion and HQ
**ALL**	9602	This data subset contains all available SNPs.

We focus on the scenarios in bold. Results of all scenarios can be found in the supplement material.

### Masking of SNPs

Imputation quality is assessed by comparing masked HQ genotypes with corresponding imputation results. For this purpose, in the scenarios *“hole filling without any reference panel”* and *“hole filling with an external reference panel”* different percentages (10, 20 and 50%) of randomly selected genotypes of HQ SNPs were masked and afterwards imputed with and without the HapMap CEU reference panel. In the scenario *“entire SNP-imputation”*, randomly selected percentages (10, 20 and 50%) of HQ SNPs were completely masked and the masked SNPs were again imputed using the HapMap CEU reference panel.

Masking of genotypes was performed in such a way that SNPs/genotypes masked in a dataset with a lower percentage of masked SNPs/genotypes were also masked in datasets with a higher percentage of masked SNPs/genotypes. Hence, the SNPs/genotypes masked in the datasets with 10% masking are also masked in all datasets of a higher percentage of masking and so on. This approach allows us to compare datasets with different percentages of missingness on the basis of an overlapping subset of masked SNPs. The different percentages of missingness were analyzed for all 16 scenarios of pre-imputation SNP filtering.

### Imputation methods

For imputation of masked SNPs, we applied the software tools MaCH1.0 [5] and IMPUTE v2.1.2 [6] following best practice guides of the authors. Formats of genotype data required by MaCH and IMPUTE were created by “fcGENE”, a format converting tool developed by our group. This tool is based on C/C++ and is freely available on Sourceforge website [28].

For imputation with MaCH1.0, 100 iterations of the Hidden Markov Model (HMM) sampler were applied with a maximum of 200 randomly chosen haplotype samples. MaCH commands are provided as supplemental material. In case of imputation with HapMap reference (HapMap3 NCBI Build 36, CEU panel), we applied the recommended two step imputation process [5],[16]. More precisely, model parameters of the underlying Hidden-Markov model were estimated by running the “greedy” algorithm. During the first step of the algorithm, both, genotyping error rates and cross-over rates were estimated. The second step exploits these parameters to impute all SNPs of the reference panel. When comparing imputation quality between the different filtering scenarios with the help of our newly proposed measures, we used the posterior probabilities contained in the MaCH output files with extension “.mlgeno”.

As recommended by IMPUTE developers [6],[17],[29],[30], we performed segmented-imputation of chromosome 22 by defining different genomic intervals approximately of size 5 MB. To avoid margin effects of chromosome segmentation, IMPUTE2 uses an internal buffer region of 250 kb on either side of the analysis interval after applying the option --buffer <250 > [17]. CEU HapMap references (HapMap3 NCBI Build 36) down-loaded from the official website of IMPUTE [17] were used for the imputation scenarios requiring a reference panel. More precisely, genetic recombination rates, reference haplotypes and the legend file were used as provided on the website.

Command options and parameters used to run MaCH and IMPUTE2 are provided in detail in the supplement material. Throughout all scenarios considered we always used the settings as described above. Reference files used for MaCH and IMPUTE2 contained exactly the same SNPs (M = 20,085).

### Assessment of imputation quality

Imputation results were assessed by two different approaches: First, we used the platform-specific measures of imputation uncertainty for each SNP as recommended by the developers of MaCH (rsq Score) and IMPUTE2 (info score). Second, we also considered two novel software-independent measures allowing a direct comparison of the observed genotype and the posterior distribution of the genotype, namely Hellinger score and the Scaled Euclidian Norm score (SEN score) as defined below.

#### *a)* SNP-wise measures implemented in MaCH (rsq) and IMPUTE (info)

MACH-rsq score equals the ratio of the empirically observed variance of the allele dosage to the expected binomial variance *p*(1-*P*) at Hardy–Weinberg equilibrium, where *p* is the observed allele frequency derived from HapMap or estimated from own data [31]. Its value tends to zero if the uncertainty of the imputation results increases. If certainty of imputed genotypes is high, this ratio is close to 1. MaCH developers recommend a threshold of at least 0.3 for reliable imputation results [16].

The IMPUTE info score is a similar measure which is based on the relative information of the observed genotype distribution compared to the complete distribution [7]. A threshold of 0.3 is recommended.

Both, MaCH-rsq and IMPUTE info score calculate uncertainty of imputation results on a SNP-wise level. Hence, they do not allow direct comparison of imputed and observed individual genotypes, and thus are of little use for the hole-filling scenarios. Due to different definitions, these two scores cannot be compared with each other directly. To overcome these limitations, two new measures were applied to assess the agreement of single observed and imputed genotypes.

#### *b)* Direct comparison of known and imputed genotypes

Suppose that any genotype of a SNP is encoded numerically with the values {0, 1, 2}, where 0 and 2 denotes homozygotes with major allele and minor allele respectively and 1 codes heterozygotes. Let *O* be the set of masked observed genotypes. Consider a single genotype *g* ∈ *O*, let *f*_1_(*g*) and *f*_2_(*g*) respectively be the trinomial probability densities of the true genotype at *g* and the genotype proposed by the imputation software, respectively. The original true genotype probability *f*_1_(*g*) can be described as

(1) $$f_{1}\left(g\right)=\left(p_{11},\;p_{12},1-\left(p_{11}+p_{12}\right)\right)$$

where

(2) $$\left(p_{11},\;p_{12},1-\left(p_{11}+p_{12}\right)\right)=\left\{\begin{array}{cc} \left({\left(1-\varepsilon \right)}^{2},\;2\varepsilon \left(1-\varepsilon \right),\;\varepsilon^{2}\right) & \mathrm{if}\;g=0\\ \left(\varepsilon \left(1-\varepsilon \right),\;{\left(1-\varepsilon \right)}^{2}+\varepsilon^{2},\;\varepsilon \left(1-\varepsilon \right)\right) & \mathrm{if}\;g=1\\ \left(\varepsilon^{2},\;2\varepsilon \left(1-\varepsilon \right),\;{\left(1-\varepsilon \right)}^{2}\right) & \mathrm{if}\;g=2 \end{array}\right)$$

and parameter *ε* is the probability of genotyping errors. Since we masked only HQ SNPs and compared them with the corresponding imputed values, we set the genotyping error rate as *ε* = 0 in the following.

Similarly, *f*_2_(*g*) can be defined as

(3) $$f_{2}\left(g\right)=\left({\tilde{p}}_{11},\;{\tilde{p}}_{12},1-\left({\tilde{p}}_{11}+{\tilde{p}}_{12}\right)\right)$$

where ${\tilde{p}}_{11}$ and ${\tilde{p}}_{12}$ are the posterior probabilities of genotypes for *g* received by the imputation process. Based on these distributions relating to original and imputed genotypes respectively, we define two scores of imputation quality as follows.

### Hellinger score

For two trinomial probability distributions *f*_1_(*g*) and *f*_2_(*g*), the Bhattacharyya coefficient which measures the amount of overlap between the two distributions [32] is defined as

(4) $$B\left(g\right)=\sum_{i=1}^{3}\sqrt{f_{1}^{i}\left(g\right)f_{2}^{i}\left(g\right)}$$

where “*i*” denotes the components of the corresponding vectors. A modified version of Bhattacharyya coefficient is the Hellinger score, which is a measure of the distance of two probability distributions [33]:

(5) $$H\left(g\right)=1-\sqrt{1-\sum_{i=1}^{3}\sqrt{f_{1}^{i}\left(g\right)f_{2}^{i}\left(g\right)}}$$

Here, the measure is equal to one if the probability distributions coincide and zero if probability vectors are perpendicular. Note that the Hellinger score is equal to one minus the Hellinger distance.

### Scaled Euclidian Norm score (SEN score)

As an alternative, one can calculate the Euclidian distance between the genotype-wise expectations of the two distributions. Let

(6) $$M^{\mathrm{obs}}=0\cdot p_{11}+1\cdot p_{12}+2\left[1-\left(p_{11}+p_{12}\right)\right]=2-\left(p_{12}+2p_{11}\right)$$

(7) $$M^{\mathrm{imp}}=0\cdot {\tilde{p}}_{11}+1\cdot {\tilde{p}}_{12}+2\left[1-\left({\tilde{p}}_{11}+{\tilde{p}}_{12}\right)\right]=2-\left({\tilde{p}}_{12}+2{\tilde{p}}_{11}\right)$$

be the expectations of the observed distribution (*f*_1_(*g*)) and the posterior distribution after imputation (*f*_2_(*g*)), then one can define the *Scaled Euclidian Norm score (SEN score)* by

(8) $$S=\left(1-\frac{{\left(M^{\mathrm{obs}}-M^{\mathrm{imp}}\right)}^{2}}{4}\right)$$

This score was constructed in a way that it ranges between 0 and 1. The greater the score, the better the corresponding genotype is imputed. In contrast to Hellinger score, SEN score only assesses the agreement of the allele doses derived from the distributions, whereas the Hellinger score can discriminate results with identical allele doses but differing genotype probabilities. Both measures are useful for assessing imputation quality of both, partially as well as completely imputed SNPs. They also allow comparisons between different imputation platforms like MaCH and IMPUTE2. To define a SNP-wise or analysis-wide measure of imputation quality, the scores can be averaged over all imputed genotypes of a certain SNP or all SNPs included in the analysis, respectively.

### Comparison between scenarios

Different scenarios of quality filtering were considered equally well suitable for imputation if resulting SNP-wise quality scores were not significantly inferior compared to the result of the best imputed scenario. We first compared the five main scenarios namely ALL, NQ, HQ, BQ and LQ. Results of additional scenarios given in Table 1 are provided in the supplement material. To allow comparisons between scenarios of different percentages of masked SNPs/genotypes, only the 10% overlapping masked SNPs and genotypes in each scenario were considered. We formally applied McNemar tests with Bonferroni-Holm correction of multiple testing (N = 5 for the main analysis and N = 16 when considering all scenarios) in order to compare percentages of well imputed genotypes defined on the basis of specified cut-offs for our newly defined quality measures. Cut-offs for Hellinger score and SEN score were chosen as 0.6 and 0.95 respectively. In our data, a cutoff 0.6 for Hellinger score ensured that the imputed best-guess genotype almost always matched the true genotype and that its posterior probability is at least 0.7. Therefore, this cut-off provides a high confidence that the best guess genotype matches the true genotype. Similarly, we observed that a SEN score greater or equal to 0.95 was indicative for the true genotype in almost every case.

Similarly we compared the performances of MaCH and IMPUTE2 by applying McNemar tests on Hellinger and SEN scores dichotomized at the specified cutoffs. Bonferroni corrected p-values with cutoff 0.05 were used to decide whether MaCH or IMPUTE2 performs better.

Performance regarding the software-specific measures MaCH-rsq and IMPUTE-info was assessed using the recommended cut-off of 0.3 for both measures.

## Results

### Hole-filling without external reference panel

In Table 2, we present the results of hole-filling without external reference for both MaCH and IMPUTE2. Shown are percentages of overlapping masked genotypes imputed with a Hellinger score ≥0.6.

**Table 2:** Imputation quality of the scenario “Hole-filling without external reference”: percentages of masked genotypes imputed with a Hellinger score ≥0.6 are presented

**Datasets**		**MACHImputation score based on 10% overlapping masked genotypes**			**IMPUTE2: Imputation score based on 10% overlapping masked genotypes**		
**Data subset name**	**#SNPs**	**10%**	**20%**	**50%**	**10%**	**20%**	**50%**
ALL	9602	93.15*	92.29*+	87.59*	***92.43***	***91.55***	***86.08+***
LQ	8472	93.23*+	92.23*	87.85*+	***92.51+***	***91.56+***	***86.00***
NQ	7923	93.08*	92.09*	87.48*	92.29	***91.36***	85.64
BQ	6337	89.82*	88.24*	79.53*	89.00	87.31	76.89
HQ	4658	89.47*	87.71*	78.56*	88.72	86.95	75.82

Datasets of different pre-imputation quality filtering were considered and different percentages of genotypes were masked. Results of the optimal imputation scenarios are described with (^+^). Results of the filtering scenarios which are not significantly inferior compared to the best scenario, are described with Italic-bold letters. An asterisk (*) indicates whether MaCH or IMPUTE2 performed significantly better in the corresponding scenario.

No single pre-processing method was optimal for all analysed scenarios, however, either ALL or LQ performed best. HQ is the dataset performing worst across all degrees of missingness. Overall, rather than the quality of SNPs, higher numbers of SNPs appeared to be associated with higher imputation quality. Only BQ (enriched with particularly worse SNPs) showed only limited improvement when comparing with the HQ dataset, although the number of SNPs in BQ was considerably larger than the number of SNPs in HQ. Therefore, our MAF filtering scenarios (LQ.MAF, HQ.MAF, HQ.MAF.HWE) are exceptions of the observed general rule that lower numbers of SNPs result in inferior imputation quality. This becomes especially apparent if comparing the scenarios HQ.CAR or BQ with the scenarios HQ.MAF or HQ.MAF.HWE. Although the first two scenarios have similar or even higher numbers of SNPs, the latter two show superior imputation results (see Additional file 1: Table S1).

Considering SEN score instead of Hellinger score provides essentially the same results (Additional file 1: Table S2). Interestingly, the currently recommended NQ filter is in no case the best option.

When comparing MaCH and IMPUTE2 in this scenario on the basis of the Hellinger Score, MaCH results were slightly better than corresponding results from IMPUTE2 (see Table 2 and Additional file 1: Table S1). However, when comparing MaCH and IMPUTE2 based on the SEN score, MaCH and IMPUTE2 showed similar performance except in a few cases where IMPUTE2 was slightly better (Additional file 1: Table S2).

### Hole-filling with external HapMap reference

Table 3 shows the results of the scenario “hole filling with external HapMap reference”. This scenario reflects basically the same trends as the previous scenario. Again, there is a clear trend towards lower imputation quality when the number of SNPs in the SNP subset becomes smaller and when the number of masked SNPs increases. Either ALL or LQ performs best and on a similar level in all scenarios. HQ again is the worst scenario across all degrees of missingness. BQ is only slightly better than HQ. Scenarios with a stringent MAF filter (HQ.MAF, HQ.MAF.HWE) showed better performance compared to the scenario HQ.CAR despite of the smaller numbers of SNPs in it. Results of other filtering scenarios can be found in Additional file 1: Tables S3 and S4.

**Table 3:** (Imputation quality of the scenario “Hole-filling with external HapMap reference”): percentages of overlapping masked genotypes imputed with good Hellinger score (≥0.6) are presented

**Datasets**		**MACH Imputation score based on 10% overlapping masked genotypes**			**IMPUTE2 Imputation score based on 10% overlapping masked genotypes**		
**Data subset name**	**#SNPs**	**10%**	**20%**	**50%**	**10%**	**20%**	**50%**
ALL	9602	94.03*+	93.44*	91.03*+	***93.12+***	***92.42+***	***89.74***
LQ	8472	94.01*	93.45*+	91.01*	***93.10***	***92.41***	***89.85+***
NQ	7923	93.83*	93.15*	90.61*	92.90	92.11	89.32
BQ	6337	90.85*	89.62*	83.34*	89.85	88.61	82.47
HQ	4658	90.47*	89.05*	81.84*	89.00	87.55	80.57

Datasets of different pre-imputation quality filtering were considered and different percentages of genotypes were masked. Results of the optimal imputation scenarios are described with (^+^). Results of the filtering scenarios which are not significantly inferior compared to the best scenario, are described with Italic-bold letters. An asterisk (*) indicates whether MaCH or IMPUTE2 performed significantly better in the corresponding scenario.

In this hole-filling scenario, MaCH performed significantly better than IMPUTE2 with Bonferroni-corrected p-values lying in between 9.68×10^−38^ and 1.45×10^−6^ when using the Hellinger Score for comparison and between 2.74×10^−32^ and 4.39×10^−5^ when using the SEN Score (see Table 3 and Additional file 1: Table S4). In all analyzed scenarios, hole-filling benefited from using an external reference panel. This improvement was statistically significant (Bonferroni corrected p-values: 1.70×10^−159^ to 6.51×10^−10^).

### Entire SNP imputation using external HapMap reference panel

In this scenario, ALL performs best for all scenarios with one exception and LQ performed on a similar level. NQ performed significantly worse than the scenario having the best score. Also, BQ performed better than HQ. MaCH often performed slightly superior compared to IMPUTE2 (see Table 4 and Additional file 1: Table S5, Bonferroni corrected p-values 1.5×10^−28^ to 0.028). Results of SEN score were similar to those of Hellinger score (Additional file 1: Table S6).

**Table 4:** (Imputation quality of the scenario ”entire SNP imputation using external HapMap reference”): percentages of overlapping masked genotypes imputed with good Hellinger score (≥0.6) are presented

**Datasets**		**MACH Imputation score based on 10% overlapping masked SNPs**			**IMPUTE2 Imputation score based on 10% overlapping masked SNPs**		
**Data subset name**	**#SNPs**	**10%**	**20%**	**50%**	**10%**	**20%**	**50%**
ALL	9602	***94.36+***	94*+	***91.63+***	***94.25+***	***93.77+***	***91.66***
LQ	8472	94.33*	93.99*	***91.61***	***94.14***	***93.68***	***91.68+***
NQ	7923	94.27	93.82*	91.33	***94.13***	93.50	91.39
BQ	6337	91.69*	90.76*	85.08	91.20	90.18	84.85
HQ	4658	91.22*	90.2*	83.64*	90.21	88.97	82.42

Datasets of different pre-imputation quality filtering were considered and different percentages of genotypes were masked. Results of the optimal imputation scenarios are described with (^+^). Results of the filtering scenarios which are not significantly inferior compared to the best scenario, are described with Italic-bold faced letters. An asterisk (*) indicates whether MaCH or IMPUTE2 performed significantly better in the corresponding scenario.

### Analysis of software specific quality scores

In Table 5 we report the imputation quality in terms of software specific quality measures, namely MaCH-rsq scores and IMPUTE-info scores. Both measures are defined on a SNP-wise level, i.e. their application is useful only for assessing the scenarios of “entire SNP imputation”. We present the percentages of SNPs imputed with good quality according to a recommended cutoff of 0.3 for both scores ([16],[17]). For MaCH, the three scenarios ALL, NQ and LQ performed similarly well. Considering IMPUTE2, all scenarios performed similarly well except for the case of 50% masking where ALL, NQ and LQ performed best. Note that MaCH-rsq and IMPUTE-info scores are defined differently and hence may not be compared directly. Indeed, using the software specific measures and cut-offs, we note that IMPUTE2 results in a higher percentage of SNPs considered as well-imputed.

**Table 5:** (Software specific quality scores for the scenario “Entire SNP imputation using external HapMap reference”): percentages of SNPs above a quality cut-off of 0.3 for both MaCH-rsq and IMPUTE-info score are provided

**Datasets**		**MaCH Imputation score observing 10% overlapping masked SNPs**			**IMPUTE2 Imputation score observing 10% overlapping masked SNPs**		
**Data subset name**	**#SNPs**	**10%**	**20%**	**50%**	**10%**	**20%**	**50%**
ALL	9602	***98.29***	***98.72+***	***98.07+***	***99.57+***	***99.57+***	***99.36***
LQ	8472	***98.29***	***98.50***	***98.07***	***99.57***	***99.57***	***99.57+***
NQ	7923	***98.50+***	***98.50***	***97.64***	***99.57***	***99.57***	***99.36***
BQ	6337	96.57	96.57	92.72	***99.14***	***98.93***	97.43
HQ	4658	96.36	96.36	91.65	***98.50***	***98.07***	96.36

Results of the optimal imputation scenarios are described with (^+^). Results of the filtering scenarios which are not significantly inferior compared to the best scenario, are described with Italic-bold letters. Note that MaCH-Rsq and IMPUTE-info scores are defined differently and cannot be compared directly.

Results of the software specific measures applying other pre-imputation filtering scenarios can be found in the supplement material (Additional file 1: Table S7). Here, all filters more or less showed similar performance.

### Comparison of quality scores

**Figure 2:**
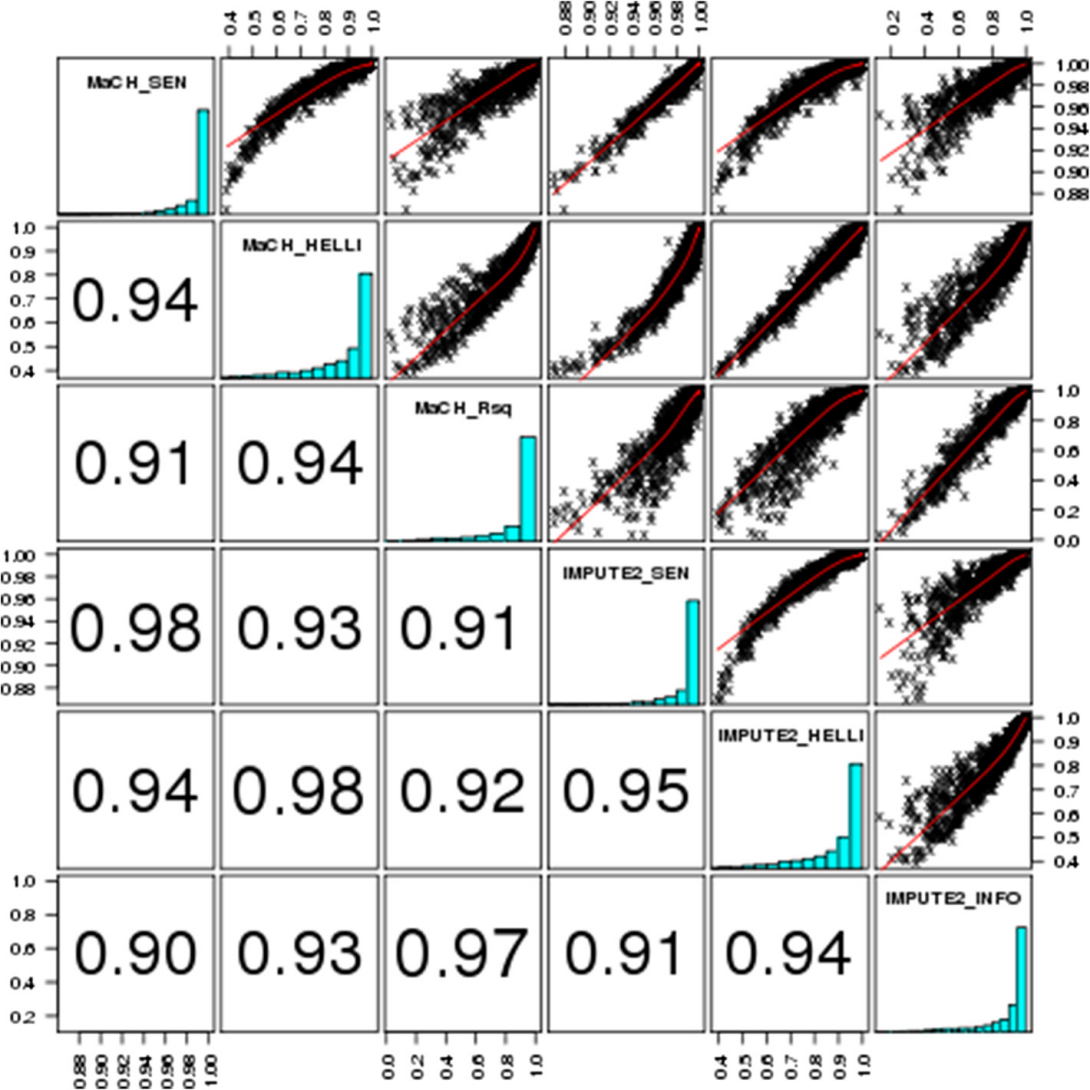
**Pairwise comparison of the analyzed measures of imputation quality.** Distribution and pair-wise correlation of SEN-scores obtained from MaCH ( MaCH_SEN) and IMPUTE (IMPUTE2_SEN), Hellinger score obtained from MaCH (MaCH_HELLI) and from IMPUTE (IMPUTE2_HELLI), MaCH Rsq-score(MaCH_Rsq) and IMPUTE-info (IMPUTE2_INFO) score are shown. We present the results for the scenario “Entire SNP imputation” without pre-filtering (“ALL”) with 50% missing SNPs. Values refer to the squared Pearson correlation.

Figure 2 shows scatter plots between different analyzed measures of imputation quality used in the present paper (scenario entire SNP imputation, dataset, “ALL”, 50% missing). We observed generally high correlations between the scores, suggesting that they capture similar information. For example, as reported ([7], supplementary information 5), correlation of MaCH- rsq and IMPUTE-info is highly linear, which is in accordance with our results.

## Discussion

In our study we addressed the question whether filtering low quality SNPs has a positive influence on the performance of the imputation algorithms MaCH and IMPUTE2. Considered filtering criteria were call rate, minor allele frequency and violation of Hardy-Weinberg equilibrium. Various degrees of stringency of these criteria were investigated. Combinations of these filtering criteria resulted in 16 different datasets comprising different numbers of SNPs (see Table 1). Three different scenarios of imputation were analyzed: hole-filling with and without an external reference panel, and imputation of entirely masked SNPs using an external reference panel. All three scenarios are of practical relevance. We introduced two novel measures for imputation performance namely Hellinger and SEN score. Both are conceptually different from established measures of imputation quality such as MACH-rsq and IMPUTE-info in the sense that the new measures allow direct comparisons of posteriori distributions with measured genotypes. This however requires a gold-standard of genotypes, which in our cases, was constructed by masking high-quality SNPs. In contrast, the software specific measures assess the uncertainty of imputation results without considering true genotype information. In consequence, it is possible that the imputed genotypes are correct but scores are low because the algorithms are uncertain about it and vice versa. To compare imputation results between different QC scenarios, we consider our measures to be a more natural choice. Furthermore, our measures can be used for any imputation platform and also on individual genotype level (i.e. for hole-filling scenarios).

The difference between Hellinger and SEN score is that the first score compares the distribution of observed and estimated genotype probabilities. The latter one measures agreement of their expectations, thereby effectively comparing agreement of the allele doses. Despite these differences, we observed strong correlations between these measures and software specific measures of imputation quality control (MaCH-rsq, IMPUTE-info).

Some researchers (e.g. [22],[34]) used the software SNPTEST to compare directly genotyped distribution and imputed distribution for each SNP. However, under the additive model, SNPTEST’s freq-addproper- info score (in the newest version, this score is called info-score only) is highly correlated with the IMPUTE-info score [7]. Therefore, we did not consider SNPTEST to assess imputation quality here.

As expected and independent of the considered quality measure, we found a clear trend towards lower imputation accuracy if the percentage of missingness increased. Furthermore, imputation quality clearly appeared to be more dependent on the number of SNPs used for imputation than on their quality. This phenomenon was observed across all scenarios and applies for all measures of imputation quality considered here. We want to point out that the dataset ALL, which ignores all kinds of SNP quality control, was never significantly outperformed by the best scenario.

In contrast, dataset BQ enriched with SNPs of considerably bad quality, as well as the dataset HQ containing the lowest number of SNPs, performed worse at approximately the same level, despite a considerably higher number of SNPs in BQ. The reason for this is that in the HQ scenario, numerous low-frequency variants are substituted by a few high-frequency variants which are more useful for imputing our masked high-frequency variants.

In consequence, accepting putatively wrong genotypes for imputation rather than thinning out possible proxies for imputation by strict quality filters appears to be the better strategy in order to achieve good imputation results. An explanation is that starting phased haplotype information at a SNP is randomly chosen if its genotypes are missing. Even if a typed SNP has lower quality, its genotypes still might provide useful information regarding haplotypes. Possibly wrong genotypes could be further corrected with the knowledge of posterior information obtained from the underlying Hidden Markov models. In consequence, our study encourages imputation without pre-filtering of SNPs or at most very restrictive filtering with cut-off levels such as those defined for the LQ dataset. However, one has to acknowledge that including SNPs with bad quality possibly requires an additional step of post-imputation quality control for typed SNPs. Hence, it is possible that typed SNPs are discarded from subsequent analyses which – if filtered prior to imputation - could be successfully imputed. This might be undesirable, especially in case of genetic meta-analyses. In consequence, there is a general conflict of interest for association analysis: Is it better to rely on measured genotypes with possible quality problems or on re-imputed genotypes with imputation uncertainty? The answer to this question is not obvious and may vary in different settings. With our work we contribute to this issue but further research is required to evaluate the consequences of both approaches for association analyses.

Our main findings are in accordance with and an extension to the study of Southam et al. [22]. Their major finding was that pre-imputation quality-filtering of SNPs results in highly similar imputation quality compared with no filtering. Our studies extend their analyses to IMPUTE2 and MaCH. Furthermore, we have shown that pre-imputation filtering can be even detrimental. We analyzed the scenarios of hole filling with and without an external reference panel not considered in [22]. Here, we showed for the first time that including an external reference is beneficial in all analyzed scenarios. However, this might depend on the genetic similarity of target and reference population, an issue which we aim to analyse in more detail in the future. Comparing MaCH and IMPUTE2 revealed frequently significantly better performance of MaCH except for a single scenario. However, this is probably a result of our moderate sample size. It has been shown [10] that the performance of MaCH is better for moderate sample sizes in contrast to larger sample sizes where IMPUTE2 performed better [6],[29]. This is explained by differences in the improvement of haplotype switch accuracies for increasing sample sizes. Interestingly, differences between software are more pronounced on the basis of Hellinger score than on the basis of SEN score.

Compared to MaCH, the software IMPUTE analyses chunks of data which allows parallelization of the imputation process. In consequence, IMPUTE generally requires less computation time than MaCH. We analysed the impact of the overlap of chunks used for imputations with IMPUTE. The default overlap for IMPTUE is 250 kB. Increasing this overlap up to 800 kB has only marginal effects on the imputation accuracy and does not explain the above mentioned observations (results not shown).

There are some limitations for our study: We analyzed a dataset of moderate size. However, moderately sized datasets are still of practical importance e.g. when combining many datasets in large meta-analysis which is common practice. In line with our results, Southam et al. [22] used a larger sample but also did not find a benefit from pre-imputation SNP filtering.

A second limitation is that we focused on common variants: The reason is that rare variant genotypes are less reliably measured by current micro-array technologies so that there is a lack of gold-standard regarding these genotypes in our study. It has been shown that imputing rare variants is difficult. Howie et al. [29] showed for example that for smaller sample sizes, the imputation accuracy of rare variants (MAF 1-3%) is considerably inferior to those for variants with MAF > 5%. Still, imputation of common variants is of general relevance for GWAS in order to improve power [1],[2].

Another limitation is that we used masked genotypes as gold standard to assess imputation accuracy instead of using measured genotypes of complementary technologies. Therefore, we used strict quality criteria for masked genotypes in order to ensure that the measured genotypes are correct. We also performed the masking randomly in order to avoid biases. An advantage of our approach is that it allows assessing different degrees of missingness in the hole-filling scenarios which is of practical relevance e.g. when combining datasets of different genotyping platforms.

In the future we aim at investigating the effect of SNP density, sample size and specific patterns of LD on imputation performance and compare it with existing studies [35]. Moreover, we also plan to investigate the impact of external reference panels on imputation quality in different ethnicities.

## Conclusion

Imputation of partially missing genotypes clearly benefits from using an external reference panel. At the cost of computation time, MaCH performed slightly better than IMPUTE2 in most of our scenarios considering a moderately sized dataset. Genotype imputation using MaCH or IMPUTE2 was robust against violations of genotype quality criteria. There is a much stronger dependence of imputation quality on percentage of missingness and numbers of SNPs in the dataset to be imputed. Therefore, SNP filtering prior to imputation is not recommended given modest quality of the data.

## Competing interests

The authors declare that they have no competing interests.

## Authors’ contributions

NRR: conceived and designed the study, method development, computational work, data analysis and paper writing, HK: data analysis, paper writing, KH: computational work, PA: data analysis, discussion, MS: conceived and designed the study, review of methods, paper writing. All authors read and approve the final manuscript.

## Additional file

## Supplementary Material

Additional file 1 Additional results and imputation commands. Click here for file
