# Supplementary material for: Impact of pre-imputation SNP-filtering on genotype imputation results
Source: BMC Genet. 2014 Aug 12;15:88. doi: 10.1186/s12863-014-0088-5 (PMC4236550; doi:10.1186/s12863-014-0088-5)
Supplement: Additional file 1 — Additional results and imputation commands. [file s12863-014-0088-5-S1.docx]

**Impact of pre-imputation SNP-Filtering on Genotype Imputation Results**

Nab Raj Roshyara^1,2^, Holger Kirsten^1,2,3,4^, Katrin Horn^1,2^, Peter Ahnert^1,2^ and Markus Scholz^1,2^

1. Institute for Medical Informatics, Statistics and Epidemiology, University of Leipzig, Haertelstrasse 16-18, 04107 Leipzig, Germany
2. LIFE Center (Leipzig Interdisciplinary Research Cluster of Genetic Factors, Phenotypes and Environment), University of Leipzig, Philipp-Rosenthal Strasse 27, 04103 Leipzig, Germany
3. Department for Cell Therapy, Fraunhofer Institute for Cell Therapy and Immunology, Perlickstrasse 1, 04103 Leipzig
4. Translational Centre for Regenerative Medicine, Universität Leipzig, Philipp-Rosenthal-Strasse 55, 04103 Leipzig

**Supplementary Material**

**1 Principal components analysis of study population in comparison with HapMapCEU:**


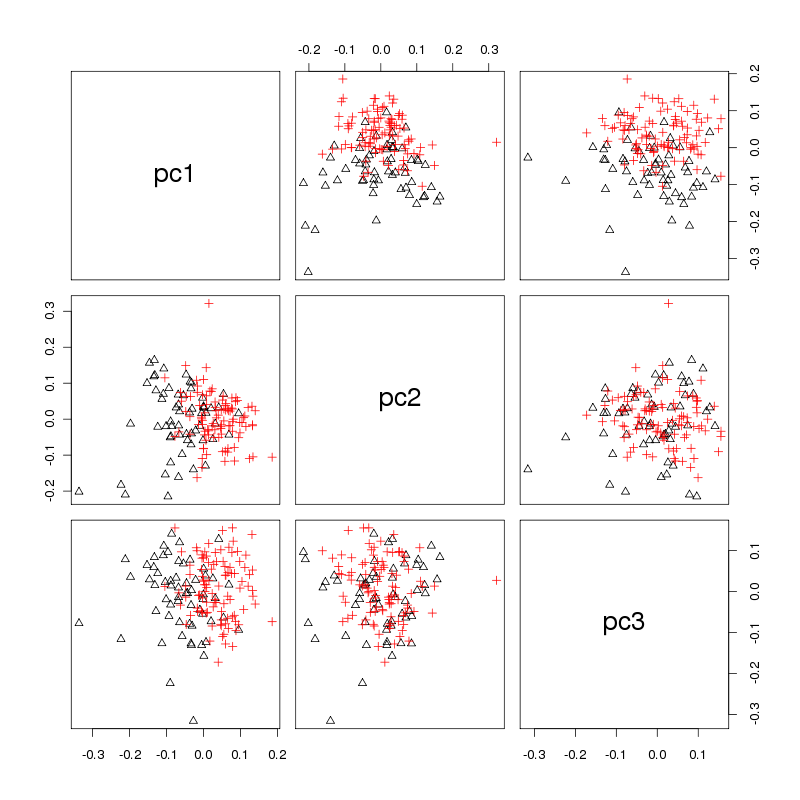


**Supplementary Figure S1:** **Principal component plot comparing all analyzed individuals with individuals from imputation reference (HapMap CEU):** Shown are the first three principal components (pc1, pc2, and pc3) as reported from EIGENSTRAT. Black triangles represent 60 individuals from HapMap CEU, red crosses represent the 100 individuals of the present study. For details, see Material and Methods section.

**2 Commands used for Imputation process:**

**2.1 Imputation without using an external reference (hole-filling without external reference):**

MaCH command for imputing masked genotypes without the use of HapMap references was applied with default settings, i.e.:

*./mach1 –p sample_chr_22.ped –d sample_chr_22.dat --states 200 --rounds 100 --geno --quality --dosage --probs --phase --mle --mldetails –prefix sample_chr_22.out*

In this command, sample_chr_22 is considered as a variable representing the different data subsets of pre-imputation quality filtering and missingness.

IMPUTE2 was also run using default parameters:

*./impute2 –phase -m hapmap3_r2_b36/genetic_map_chr22_combined_b36.txt \*

*-g sample_chr_22 .gens -strand_g sample_chr_22.strand -int lower_int upper_int \*

*-Ne 11418 -call_thresh 0.9 -pgs -o sample_ chr_22.out*

The two numbers lower_int and upper_int used in IMPUTE command are the base pair positions which define the boundaries of the genomic interval used for genotype inference.

**2.2 Imputation using HapMap References (hole-filling scenario with HapMap reference panel, entire SNP imputation):**

For imputation with MaCH, we applied the following two stepwise process in order to impute both, masked genotypes and completely masked SNPs.

**Step1:** *./mach1 -p sample_ chr_22.ped -d sample_chr_22.dat -s hm3_r2_b36_fwd.CEU.chr22.snps\*

*-h hm3_r2_b36_fwd.CEU.chr22.hap --greedy -r 100 –prefix sample_chr_22_step1*

**Step2:** *./mach1 -p sample_chr_22.ped -d sample_chr_22.dat -s hm3_r2_b36_fwd.CEU.chr22.snps \*

*-h hm3_r2_b36_fwd.CEU.chr22.hap --crossover sample_chr_22_step1.rec \*

*--errormap sample_chr_22_step1.erate --greedy --geno --quality --dosage --probs --phase \*

*--mle --mldetails --prefix sample_ chr_22_step2*

In the first step, both, genotyping error rates and crossover rates are calculated. Using these parameters. In the second step, all SNPs of the reference panel are imputed. When analysing imputation quality across different data subsets, we used the posterior probabilities for two of the three genotypes at each SNP and for each individual as contained in MaCH output files with extension “.mlgeno”.

In order to perform imputations with IMPUTE2 using HapMap references, we used the following command:

*./impute2 -m hapmap3_r2_b36/genetic_map_chr22_combined_b36.txt \*

*-h hapmap3_r2_b36/hapmap3.r2.b36.all.chr22.haps\*

*–l hapmap3_r2_b36/hapmap3.r2.b36.all.chr22.legend -g sample_chr_22.gens \*

*-strand_g sample_chr_22.strand -int lower_int upper_int -Ne 11418 -call_thresh 0.9 \*

*-pgs -o sample_chr_22.out*

After completion of the imputation process, we analysed the quality of imputed genotypes using the three posterior probabilities that are given for each genotype and for each individual as contained in the imputation output files.

**3 Results of additional pre-imputation quality filters:**

In the following tables we present the results of additional pre-imputation quality filters for the three imputation scenarios considered in the main paper.

**Structure of the Tables:** Rows of tables represent different pre-imputation quality filters ordered by the number of SNPs contained in the resulting data subsets. The first and second columns contain the names of data subsets described in Table 1 of the main paper and the total number of SNPs contained in the corresponding data subsets, respectively. The other columns contain the imputation quality scores for a different number of masked SNPs or genotypes and for the two imputation software considered. The cell with the best score in any particular column represents the best scenario and is marked by dark background. Scenarios with non-significantly inferior imputation quality are marked by light grey background. Quality scores were compared with the Mc-Nemar test. Only the 10% overlapping masked SNPs or genotypes (that are masked in all scenarios) were assessed for imputation quality. P-values were corrected for multiple testing using the method of Bonferroni-Holm.

**3.1 Results of the scenario “Hole filling without external reference”**

| **Datasets** | | **MACH Imputation score based on 10% overlapping masked genotypes** | | | **IMPUTE Imputation score based on 10% overlapping masked genotypes** | | |
| --- | --- | --- | --- | --- | --- | --- | --- |
| **Data subset name** | **#SNPs** | **10%** | **20%** | **50%** | **10%** | **20%** | **50%** |
| ALL | 9602 | 93.15* | 92.29* | 87.59* | 92.43 | 91.55 | 86.08 |
| LQ.HWE | 9574 | 93.17* | 92.3* | 87.54* | 92.51 | 91.66 | 86.15 |
| NQ.HWE | 9547 | 93.3* | 92.27* | 87.68* | 92.57 | 91.64 | 86.11 |
| HQ.HWE | 9450 | 93.13* | 92.3* | 87.89* | 92.48 | 91.48 | 85.98 |
| NQ.CAR | 9194 | 93.12* | 92.24* | 87.45* | 92.28 | 91.35 | 85.65 |
| LQ.MAF | 8520 | 93.25* | 92.34* | 87.54* | 92.51 | 91.61 | 86.04 |
| LQ.MAF.HWE | 8492 | 93.19* | 92.43* | 87.73* | 92.50 | 91.59 | 86.25 |
| LQ | 8472 | 93.23* | 92.23* | 87.85* | 92.51 | 91.56 | 86.00 |
| NQ.MAF | 8310 | 93.2* | 92.39* | 87.71* | 92.43 | 91.52 | 85.78 |
| NQ.MAF.HWE | 8255 | 93.21* | 92.37* | 87.86* | 92.56 | 91.65 | 86.21 |
| NQ | 7923 | 93.08* | 92.09* | 87.48* | 92.29 | 91.36 | 85.64 |
| HQ.CAR | 7148 | 90.7* | 89.29* | 80.76* | 89.91 | 88.31 | 78.62 |
| HQ.MAF | 6344 | 92.52* | 91.45* | 86.66* | 91.61 | 90.74 | 84.71 |
| BQ | 6337 | 89.82* | 88.24* | 79.53* | 89.00 | 87.31 | 76.89 |
| HQ.MAF.HWE | 6261 | 92.5* | 91.37* | 86.25* | 91.68 | 90.44 | 84.74 |
| HQ | 4658 | 89.47* | 87.71* | 78.56* | 88.72 | 86.95 | 75.82 |

**Supplementary Table S1: (Imputation quality based on Hellinger score of 10% overlapping masked genotypes in 16 different scenarios of pre-imputation quality control for the scenario “hole filling without external reference”):** Percentages of the overlapping masked genotypes imputed with Hellinger score greater than or equal to 0.6 are presented. Datasets of different pre-imputation quality filtering were considered and different percentages of genotypes were masked. Imputations were performed with either MaCH or IMPUTE2. Scenarios with optimal imputation results are marked by dark background. Light grey background marks results which are not significantly inferior compared to the best scenario. An asterisk (*) indicates whether MaCH or IMPUTE2 performed significantly better in the corresponding scenario (Bonferroni corrected).

| **Datasets** | | **MACH Imputation score based on 10% overlapping masked genotypes** | | | **IMPUTE Imputation score based on 10% overlapping masked genotypes** | | |
| --- | --- | --- | --- | --- | --- | --- | --- |
| Data subset name | #SNPs | 10% | 20% | 50% | 10% | 20% | 50% |
| ALL | 9602 | 95.09 | 94.45 | 90.76 | 95.27 | 94.76* | 91.25* |
| LQ.HWE | 9574 | 95.16 | 94.45 | 90.81 | 95.34 | 94.74* | 91.16 |
| NQ.HWE | 9547 | 95.26 | 94.52 | 90.90 | 95.33 | 94.75 | 91.31* |
| HQ.HWE | 9450 | 95.08 | 94.58 | 91.08 | 95.25 | 94.65 | 91.29 |
| NQ.CAR | 9194 | 95.06 | 94.47 | 90.78 | 95.24 | 94.61 | 91.00 |
| LQ.MAF | 8520 | 95.21 | 94.58 | 90.75 | 95.22 | 94.70 | 91.25* |
| LQ.MAF.HWE | 8492 | 95.13 | 94.60 | 90.98 | 95.32 | 94.74 | 91.28 |
| LQ | 8472 | 95.23 | 94.47 | 91.06 | 95.30 | 94.78* | 91.05 |
| NQ.MAF | 8310 | 95.13 | 94.63 | 90.81 | 95.24 | 94.61 | 91.08 |
| NQ.MAF.HWE | 8255 | 95.13 | 94.49 | 90.87 | 95.4* | 94.68 | 91.25 |
| NQ | 7923 | 95.04 | 94.35 | 90.83 | 95.18 | 94.64* | 90.86 |
| HQ.CAR | 7148 | 93.58 | 92.52 | 86.11 | 93.58 | 92.62 | 86.55 |
| HQ.MAF | 6344 | 94.54 | 93.85 | 90.19 | 94.73 | 94.07 | 90.24 |
| BQ | 6337 | 92.91 | 91.61 | 85.29 | 93.04 | 92* | 85.33 |
| HQ.MAF.HWE | 6261 | 94.61 | 93.84 | 89.79 | 94.68 | 93.93 | 90.19 |
| HQ | 4658 | 92.61 | 91.36 | 84.20 | 92.86 | 91.66 | 84.60 |

**Supplementary Table S2: (Imputation quality based on SEN score of 10% overlapping masked genotypes in 16 different scenarios of pre-imputation quality control for the scenario “hole filling without external reference”):** Percentages of the overlapping masked genotypes imputed with a SEN score greater than or equal to 0.95 are presented. Datasets of different pre-imputation quality filtering were considered and different percentages of genotypes were masked. Imputations were performed with either MaCH or IMPUTE2. Scenarios with optimal imputation results are marked by dark background. Light grey background marks results which are not significantly inferior compared to the best scenario. An asterisk (*) indicates whether MaCH or IMPUTE2 performed significantly better in the corresponding scenario (Bonferroni corrected).

**3.2 Results for the scenario “Hole filling with external HapMap reference”**

| **Datasets** | | **MACH Imputation score based on 10% overlapping masked genotypes** | | | **IMPUTE Imputation score based on 10% overlapping masked genotypes** | | |
| --- | --- | --- | --- | --- | --- | --- | --- |
| Data subset name | #SNPs | 10% | 20% | 50% | 10% | 20% | 50% |
| ALL | 9602 | 94.03* | 93.44* | 91.03* | 93.12 | 92.42 | 89.74 |
| LQ.HWE | 9574 | 94.04* | 93.46* | 91.03* | 93.10 | 92.39 | 89.82 |
| NQ.HWE | 9547 | 94.02* | 93.44* | 90.99* | 93.12 | 92.45 | 89.84 |
| HQ.HWE | 9450 | 94* | 93.38* | 90.95* | 93.06 | 92.45 | 89.78 |
| NQ.CAR | 9194 | 93.79* | 93.18* | 90.65* | 92.88 | 92.15 | 89.46 |
| LQ.MAF | 8520 | 94.06* | 93.46* | 91.06* | 93.03 | 92.33 | 89.79 |
| LQ.MAF.HWE | 8492 | 94.02* | 93.48* | 91.01* | 93.12 | 92.31 | 89.76 |
| LQ | 8472 | 94.01* | 93.45* | 91.01* | 93.10 | 92.41 | 89.85 |
| NQ.MAF | 8310 | 94.06* | 93.48* | 91.01* | 92.98 | 92.37 | 89.72 |
| NQ.MAF.HWE | 8255 | 94.03* | 93.46* | 91.01* | 93.09 | 92.33 | 89.78 |
| NQ | 7923 | 93.83* | 93.15* | 90.61* | 92.90 | 92.11 | 89.32 |
| HQ.CAR | 7148 | 91.62* | 90.5* | 84.71* | 90.67 | 89.47 | 83.97 |
| HQ.MAF | 6344 | 93.29* | 92.62* | 89.52* | 91.98 | 91.19 | 87.97 |
| BQ | 6337 | 90.85* | 89.62* | 83.34* | 89.85 | 88.61 | 82.47 |
| HQ.MAF.HWE | 6261 | 93.24* | 92.54* | 89.34* | 91.89 | 91.08 | 87.91 |
| HQ | 4658 | 90.47* | 89.05* | 81.84* | 89.00 | 87.55 | 80.57 |

**Supplementary Table S3: (Imputation quality based on Hellinger score of 10% overlapping masked genotypes in 16 different scenarios of pre-imputation quality control for the scenario “hole filling with external HapMap reference”):** Percentages of the overlapping masked genotypes imputed with Hellinger score greater than or equal to 0.6 are presented. Datasets of different pre-imputation quality filtering were considered and different percentages of genotypes were masked. Imputations were performed with either MaCH or IMPUTE2. Scenarios with optimal imputation results are marked by dark background. Light grey background marks results which are not significantly inferior compared to the best scenario. An asterisk (*) indicates whether MaCH or IMPUTE2 performed significantly better in the corresponding scenario (Bonferroni corrected).

| **Datasets** | | **MACH Imputation score based on 10% overlapping masked genotypes** | | | **IMPUTE Imputation score based on 10% overlapping masked genotypes** | | |
| --- | --- | --- | --- | --- | --- | --- | --- |
| Data subset name | #SNPs | 10% | 20% | 50% | 10% | 20% | 50% |
| ALL | 9602 | 96.06* | 95.71* | 94.22* | 95.64 | 95.14 | 93.51 |
| LQ.HWE | 9574 | 96.03* | 95.68* | 94.26* | 95.60 | 95.17 | 93.52 |
| NQ.HWE | 9547 | 96* | 95.67* | 94.24* | 95.60 | 95.18 | 93.53 |
| HQ.HWE | 9450 | 96.02* | 95.67* | 94.15* | 95.57 | 95.14 | 93.45 |
| NQ.CAR | 9194 | 95.9* | 95.55* | 94.01* | 95.45 | 95.00 | 93.17 |
| LQ.MAF | 8520 | 96.05* | 95.72* | 94.26* | 95.56 | 95.19 | 93.57 |
| LQ.MAF.HWE | 8492 | 96.04* | 95.71* | 94.24* | 95.60 | 95.15 | 93.41 |
| LQ | 8472 | 96.04* | 95.71* | 94.24* | 95.60 | 95.16 | 93.47 |
| NQ.MAF | 8310 | 96.05* | 95.72* | 94.23* | 95.60 | 95.09 | 93.42 |
| NQ.MAF.HWE | 8255 | 96.02* | 95.7* | 94.24* | 95.62 | 95.16 | 93.50 |
| NQ | 7923 | 95.94* | 95.53* | 93.98* | 95.48 | 94.93 | 93.11 |
| HQ.CAR | 7148 | 94.7* | 94.02* | 90.71* | 94.10 | 93.28 | 89.76 |
| HQ.MAF | 6344 | 95.6* | 95.19* | 93.25* | 94.89 | 94.40 | 92.31 |
| BQ | 6337 | 94.22* | 93.48* | 89.79* | 93.59 | 92.72 | 88.50 |
| HQ.MAF.HWE | 6261 | 95.54* | 95.12* | 93.16* | 94.93 | 94.36 | 92.23 |
| HQ | 4658 | 94* | 93.16* | 89.11* | 93.06 | 92.17 | 87.55 |

**Supplementary Table S4: (Imputation quality based on SEN score of 10% overlapping masked genotypes in 16 different scenarios of pre-imputation quality control for the scenario “hole filling with external HapMap reference”):** Percentages of the overlapping masked genotypes imputed with a SEN score greater than or equal to 0.95 are presented. Datasets of different pre-imputation quality filtering were considered. Different percentages of genotypes were masked. Imputations were performed with either MaCH or IMPUTE2. Scenarios with optimal imputation results are marked by dark background. Light grey background marks results which are not significantly inferior compared to the best scenario. An asterisk (*) indicates whether MaCH or IMPUTE2 performed significantly better in the corresponding scenario (Bonferroni corrected).

**3.3 Results of the scenario “entire SNP imputation with external HapMap reference”:**

| **Datasets** | | **MACH Imputation score based on 10% overlapping masked SNPs** | | | **IMPUTE Imputation score based on 10% overlapping masked SNPs** | | |
| --- | --- | --- | --- | --- | --- | --- | --- |
| Data subset name | #SNPs | 10% | 20% | 50% | 10% | 20% | 50% |
| ALL | 9602 | 94.36 | 94* | 91.63 | 94.25 | 93.77 | 91.66 |
| LQ.HWE | 9574 | 94.32 | 93.98 | 91.60 | 94.19 | 93.85 | 91.78 |
| NQ.HWE | 9547 | 94.33 | 93.97* | 91.68 | 94.18 | 93.77 | 91.84 |
| HQ.HWE | 9450 | 94.29 | 93.95* | 91.58 | 94.21 | 93.76 | 91.71 |
| NQ.CAR | 9194 | 94.29 | 93.83* | 91.36 | 94.17 | 93.64 | 91.42 |
| LQ.MAF | 8520 | 94.37* | 94.01* | 91.66 | 94.09 | 93.67 | 91.63 |
| LQ.MAF.HWE | 8492 | 94.30 | 93.94 | 91.62 | 94.14 | 93.79 | 91.63 |
| LQ | 8472 | 94.33* | 93.99* | 91.61 | 94.14 | 93.68 | 91.68 |
| NQ.MAF | 8310 | 94.33* | 94.01* | 91.67* | 94.12 | 93.62 | 91.43 |
| NQ.MAF.HWE | 8255 | 94.29 | 93.99* | 91.63 | 94.13 | 93.72 | 91.61 |
| NQ | 7923 | 94.27 | 93.82* | 91.33 | 94.13 | 93.50 | 91.39 |
| HQ.CAR | 7148 | 92.28* | 91.39* | 85.97 | 92.01 | 91.17 | 86.62* |
| HQ.MAF | 6344 | 93.78* | 93.32* | 90.42* | 93.12 | 92.42 | 89.54 |
| BQ | 6337 | 91.69* | 90.76* | 85.08 | 91.20 | 90.18 | 84.85 |
| HQ.MAF.HWE | 6261 | 93.75* | 93.24* | 90.36* | 93.07 | 92.42 | 89.36 |
| HQ | 4658 | 91.22* | 90.2* | 83.64* | 90.21 | 88.97 | 82.42 |

**Supplementary Table S5: (Imputation quality based on Hellinger score of 10% overlapping masked SNPs in 16 different scenarios of pre-imputation quality control for the scenario “entire SNP imputation with external HapMap reference”):** Percentages of the overlapping masked SNPs imputed with Hellinger score greater than or equal to 0.6 are presented. Datasets of different pre-imputation quality filtering were considered and different percentages of SNPs were masked. Imputations were performed with either MaCH or IMPUTE2. Scenarios with optimal imputation results are marked by dark background. Light grey background marks results which are not significantly inferior compared to the best scenario. An asterisk (*) indicates whether MaCH or IMPUTE2 performed significantly better in the corresponding scenario (Bonferroni corrected).

| **Datasets** | | **MACH Imputation score based on 10% overlapping masked SNPs** | | | **IMPUTE Imputation score based on 10% overlapping masked SNPs** | | |
| --- | --- | --- | --- | --- | --- | --- | --- |
| Data subset name | #SNPs | 10% | 20% | 50% | 10% | 20% | 50% |
| ALL | 9602 | 96.24 | 95.97 | 94.55 | 96.22 | 95.95 | 94.68 |
| LQ.HWE | 9574 | 96.24 | 96.00 | 94.54 | 96.23 | 95.99 | 94.60 |
| NQ.HWE | 9547 | 96.22 | 95.95 | 94.56 | 96.22 | 95.97 | 94.72 |
| HQ.HWE | 9450 | 96.20 | 95.96 | 94.51 | 96.27 | 95.98 | 94.69 |
| NQ.CAR | 9194 | 96.22 | 95.92 | 94.37 | 96.24 | 95.84 | 94.53 |
| LQ.MAF | 8520 | 96.25 | 95.96 | 94.56 | 96.20 | 95.86 | 94.58 |
| LQ.MAF.HWE | 8492 | 96.20 | 95.95 | 94.55 | 96.26 | 95.92 | 94.63 |
| LQ | 8472 | 96.22 | 95.96 | 94.52 | 96.16 | 95.96 | 94.66 |
| NQ.MAF | 8310 | 96.20 | 95.97 | 94.54 | 96.19 | 95.94 | 94.55 |
| NQ.MAF.HWE | 8255 | 96.19 | 95.96 | 94.56 | 96.23 | 95.94 | 94.55 |
| NQ | 7923 | 96.19 | 95.89 | 94.34 | 96.19 | 95.83 | 94.39 |
| HQ.CAR | 7148 | 95.02 | 94.48 | 91.32 | 94.91 | 94.34 | 91.37 |
| HQ.MAF | 6344 | 95.9* | 95.57* | 93.77* | 95.60 | 95.20 | 93.30 |
| BQ | 6337 | 94.58 | 93.98 | 90.69* | 94.34 | 93.72 | 90.16 |
| HQ.MAF.HWE | 6261 | 95.88 | 95.5* | 93.75* | 95.62 | 95.19 | 93.30 |
| HQ | 4658 | 94.34* | 93.71* | 89.91* | 93.80 | 93.01 | 88.86 |

**Supplementary Table S6: (Imputation quality based on SEN score of 10% overlapping masked SNPs in 16 different scenarios of pre-imputation quality control for the scenario “entire SNP imputation with external HapMap reference”):** Percentages of the overlapping masked SNPs imputed with SEN score greater than or equal to 0.95 are presented and datasets of different pre-imputation quality filtering were considered. Different percentages of SNPs were masked. Imputations were performed with either MaCH or IMPUTE2. Scenarios with optimal imputation results are marked by dark background. Light grey background marks results which are not significantly inferior compared to the best scenario. An asterisk (*) indicates whether MaCH or IMPUTE2 performed significantly better in the corresponding scenario (Bonferroni corrected).

| **Datasets** | | **MaCH-rsq score based on 10% overlapping masked SNPs** | | | **IMPUTE-info score score based on 10% overlapping masked SNPs** | | |
| --- | --- | --- | --- | --- | --- | --- | --- |
| Data subset name | SNPs No | 10% | 20% | 50% | 10% | 20% | 50% |
| ALL | 9602 | 98.29 | 98.72 | 98.07 | 99.57 | 99.57 | 99.36 |
| LQ.HWE | 9574 | 98.50 | 98.72 | 98.07 | 99.57 | 99.57 | 99.57 |
| NQ.HWE | 9547 | 98.29 | 98.72 | 98.07 | 99.57 | 99.79 | 99.57 |
| HQ.HWE | 9450 | 98.50 | 98.50 | 97.86 | 100.00 | 99.57 | 99.36 |
| NQ.CAR | 9194 | 98.50 | 98.50 | 97.86 | 99.57 | 99.57 | 99.57 |
| LQ.MAF | 8520 | 98.29 | 98.29 | 97.86 | 99.57 | 99.57 | 99.36 |
| LQ.MAF.HWE | 8492 | 98.29 | 98.72 | 97.86 | 99.57 | 99.57 | 99.57 |
| LQ | 8472 | 98.29 | 98.50 | 98.07 | 99.57 | 99.57 | 99.57 |
| NQ.MAF | 8310 | 98.29 | 98.50 | 98.07 | 99.57 | 99.79 | 99.57 |
| NQ.MAF.HWE | 8255 | 98.50 | 98.50 | 98.07 | 99.57 | 99.57 | 99.57 |
| NQ | 7923 | 98.50 | 98.50 | 97.64 | 99.57 | 99.57 | 99.36 |
| HQ.CAR | 7148 | 96.79 | 96.79 | 93.36 | 99.57 | 99.14 | 98.72 |
| HQ.MAF | 6344 | 97.86 | 97.86 | 96.57 | 99.14 | 98.93 | 98.50 |
| BQ | 6337 | 96.57 | 96.57 | 92.72 | 99.14 | 98.93 | 97.43 |
| HQ.MAF.HWE | 6261 | 97.86 | 97.86 | 96.57 | 98.93 | 98.93 | 98.72 |
| HQ | 4658 | 96.36 | 96.36 | 91.65 | 98.50 | 98.07 | 96.36 |

**Supplementary Table S7: (Imputation quality of 10% overlapping masked SNPs in 16 different scenarios of pre-imputation quality control for the scenario “entire SNP imputation with HapMap reference”):** Percentages of SNPs with MaCH-rsq or IMPUTE-info score greater than or equal to 0.3 are presented. Datasets of different pre-imputation quality filtering were considered. Different percentages of SNPs were masked. Scenarios with optimal imputation results are marked by dark grey background. Light grey background marks results which are not significantly inferior compared to the best scenario.
